# Drunk and Disorganised: Relationships between Bar Characteristics and Customer Intoxication in European Drinking Environments 

**DOI:** 10.3390/ijerph9114068

**Published:** 2012-11-12

**Authors:** Karen Hughes, Zara Quigg, Mark A. Bellis, Amador Calafat, Ninette van Hasselt, Matej Kosir, Lotte Voorham, Ferry X. Goossens, Mariangels Duch, Montse Juan

**Affiliations:** 1 Centre for Public Health, Liverpool John Moores University, Henry Cotton Building, 15-21 Webster Street, Liverpool L3 2ET, UK; Email: z.a.quigg@ljmu.ac.uk (Z.Q.); m.a.bellis@ljmu.ac.uk (M.A.B.); 2 European Institute of Studies on Prevention (IREFREA), Rambla 15, 07003 Palma de Mallorca, Spain; Email: irefrea@irefrea.org (A.C.); mduch@irefrea.org (M.D.); irefrea@cop.es (M.J.); 3 Trimbos-instituut, Da Costakade 45, 3521 VS Utrecht, Netherlands; Email: nhasselt@trimbos.nl (N.V.H.); lvoorham@trimbos.nl (L.V.); fgoossens@trimbos.nl (F.X.G.); 4 Institute for Research and Development “Utrip”, Trubarjeva cesta 13, SI-1290 Grosuplje, Llubljana, Slovenia; Email: info@institut-utrip.si

**Keywords:** alcohol, intoxication, drinking environments, prevention, harm reduction

## Abstract

Preventing alcohol-related harm in drinking environments is a growing international priority. Factors relating to the physical, social and staffing environments in bars can contribute to increased alcohol consumption and harm. Understanding the relationships between such factors and intoxication in European drinking environments is critical to developing appropriate interventions. We undertook a quantitative observational study in 60 bars in four European cities, in The Netherlands, Slovenia, Spain and the UK (n = 237 observational visits). Using a structured observational schedule, researchers recorded characteristics of the bar environment and rated customer intoxication levels. All physical bar characteristics showed associations with intoxication before interactions between them were controlled for. Hierarchical modelling found significant independent associations between intoxication and use of plastic glassware, promotion of non-alcoholic drinks (often energy drinks), permissive environments, poor washroom facilities, the presence of a dance floor, customer sexual activity/competitiveness and later observational time. Findings suggest that prevention efforts should focus on raising and enforcing managerial standards in bars. While harm reduction measures such as plastic glassware are often promoted for high risk bars, such measures are inadequate to address public health concerns and insufficient to demonstrate social responsibility.

## 1. Introduction

Preventing alcohol-related harm in drinking environments is a growing international priority. The World Health Organization’s global alcohol strategy [1] identifies drinking environments as key settings for interventions to reduce the negative consequences of alcohol. Suggested policy options include measures to regulate drinking contexts to minimise harm and implement management policies regarding responsible beverage service. Equally, the European alcohol action plan [2] recognises the importance of bar environments in increasing or preventing alcohol-related problems, and suggests the development of guidelines and standards for the design of drinking premises, server training and the monitoring and enforcement of licensing laws. This focus on drinking environments is backed up by a strong body of research showing that high levels of alcohol use and related problems occur in and around bars and nightclubs [3,4,5,6]. Binge drinking and intoxication are common among nightlife users [7], and studies consistently associate higher densities of drinking premises with greater alcohol-related harm, particularly violence [8,9,10]. The presence of intoxicated customers in bars increases risks of such harm [11,12,13], highlighting the need for prevention measures to focus on reducing intoxication [13]. 

Alcohol-related harm is often concentrated in specific problematic venues [14]. This can relate to management choices in such venues, including those around bar design, staff practice, entertainment provision and type of clientele targeted [15,16]. Recognition of the importance of bar environments in promoting or preventing alcohol-related problems has driven research to identify characteristics of bars that can contribute to alcohol-related harm [15,17,18,19]; and consequently that can be moderated to prevent harm [11]. A review of these studies identified numerous factors that have emerged as important in predicting greater alcohol use and harm, including poor cleanliness, crowding, loud music, and a permissive environment (*i.e.*, tolerance towards anti-social behaviour) [20]. However, most studies identified had been conducted in non-European settings, and most had focused on alcohol-related harm rather than intoxication. Thus, there is currently a lack of knowledge to inform the development of venue-focused interventions in European drinking environments. To address this gap, we undertook a quantitative observational study in youth-oriented bars in four European cities. 

## 2. Methods

The study took place in Utrecht (the Netherlands), Ljubljana (Slovenia), Palma de Mallorca (Spain) and Liverpool (UK) (for further information on each city see [7]). In each city, 15 venues popular with young people were identified for inclusion in the study, providing a sample of 60 venues. Two strategies were used to identify venues. In Liverpool, Ljubljana and Utrecht, researchers liaised with relevant authorities to identify all youth-focused bars and categorise these into low, medium or high risk premises based on local data/knowledge of alcohol-related harm. From each group, five premises were randomly selected for the study. In Palma, low, medium and high risk venues were selected based on consultation with local nightlife users.

The observation schedule used to assess premises and the implementation method was based on that developed by Graham *et al*. [17]. The schedule comprised a range of scale variables and other questions designed to measure aspects of the bar environment (see Appendix Table A1). The original schedule was altered slightly following a research meeting to tailor it to contemporary bar environments in Europe; some items were removed (e.g., pool table atmosphere) and some added (e.g., the price of certain drinks). Research leads from each country undertook a training session to develop consistency in implementing the observational visits, completing the schedule and recognizing and rating intoxication. For the latter, focus was placed on observational indicators that researchers could use to recognise different stages of intoxication, including changes in drinkers’ behaviour, appearance and coordination. The training also included a test bar observation, with research leads completing the schedule independently after the visit and comparing and discussing ratings at a meeting the following day. Each research lead then recruited field researchers in their country and repeated the training programme. 

In each city, covert one-hour observational visits were undertaken to each venue during peak opening hours on four separate occasions, with days and times of visits varied for each venue. Each observational visit was conducted by a mixed gender pair. Observations took place on Thursday, Friday and Saturday nights (September to December 2010) between 10 p.m. and 5 a.m., with study timings dependent upon local nightlife activity. In Utrecht, researchers were unable to undertake a fourth visit to two premises. Thus, 238 observational visits were undertaken. During observations, researchers were instructed to position themselves in areas with good visibility and to move around to ensure they observed all parts of the venue. They were requested to: behave as customers (being permitted to consume one alcoholic drink); dress in clothing appropriate to the venue; remain as inconspicuous as possible; and avoid unnecessary interaction with other customers. Covert note taking was permitted on mobile phones. Following each visit, researchers independently completed the observational schedule. Paired schedules were later checked at a research meeting with fieldworkers and research leads, with differences between the two schedules discussed and consensus met. Thus, each observation resulted in a single completed schedule. Ethical approval for the study was obtained from Liverpool John Moores University research ethics committee in the UK.

Analysis used SPSS version 17. The primary dependent variable was “intoxication level of people in the venue”, measured on a scale of 0 (no sign of intoxication) to 9 (everyone is drunk). This scale had not been completed for one observation in Utrecht and this visit was excluded from analysis (n = 237 visits). For environmental characteristics, measures that used a 0–9 scale were entered as continuous variables with most other data items dichotomised into categorical variables (see Appendix Table A1). Two measures recorded as percentages (customers dancing, seating) were converted into scale variables (see Appendix Table A1). Data completeness was high across all variables (>98% with the exception of individual drink prices; 98% of visits provided at least one drink price and 67% provided all four drink prices). Missing values were imputed as the city mean for scale variables or the venue norm for dichotomous variables. 

Bars can vary their operation at different times and consequently each visit was used as a separate observation in analysis rather than an average being calculated for a venue. City level comparisons of environmental characteristics recorded at each visit used chi squared and ANOVA. For multivariate analysis, scale variables that were highly correlated (r > 0.50) were combined in composite scales (see Appendix Table A1). Analysis used hierarchical modelling (linear mixed modelling) with venue as the unit of observation. All variables were initially input individually to identify associations with intoxication. Variables were then entered into six separate multivariate models relating to: (1) venue entrance; (2) physical environment; (3) bar activities; (4) alcohol and food service; (5) venue staff; and (6) customer factors. Five additional contextual variables were analysed: city; observation time (an equal split between earlier/later observations in each city); number of customers in the premise (>100 or not at the busiest time); whether police were outside the venue during the observation (which may have affected staff/customer behaviour); and whether the venue had an outdoor drinking area. Variables with independent relationships with intoxication ratings within each model were entered into the final models. 

## 3. Results

Table 1 and Table 2 show the distribution of environmental characteristics recorded during observational visits by city. There were significant differences between cities for most characteristics. For example, door staff were present during fewer observational visits in Ljubljana than in other cities, while alcoholic drink promotions were most commonly seen in Liverpool (Table 1). Observers in Utrecht recorded the highest mean rating on the cleanliness scale (*i.e.*, lower levels of cleanliness). In Palma, most observations identified high alcohol content drinks (predominantly spirits) to be the dominant drink types consumed, whereas in Utrecht low alcohol content drinks (e.g., lager) dominated. Table 1 shows the mean prices of drinks purchased across cities. The mean price of a bottle of lager, for example, ranged from €2.28 in Utrecht to €4.18 in Palma. In general, observations in Palma recorded fewer bar staff per customer and more female and older bar staff (Table 2). Across all customer behaviour variables, mean ratings were lowest in Ljubljana although differences between cities were only significant for sexual competition and rowdiness. There were no significant differences between cities in mean ratings of customer intoxication (Liverpool and Utrecht 4.0, Palma 3.7, Ljubljana 3.5, *P* = 0.313).

At the initial stage of hierarchical modelling, significant associations were seen between customer intoxication ratings and all physical environment characteristics, as well as most venue entry characteristics (Table 3). For bar activities, only the presence of a dance floor was associated with higher intoxication ratings, while for alcohol and food service, non-alcoholic (soft) drink promotions and plastic glassware were associated with higher intoxication ratings, and table and food service with lower ratings. For venue staff, the presence of glass collectors, poorer staff monitoring, staff attitude, staff boundaries and higher levels of permissiveness were associated with intoxication. Younger clientele and higher levels of customer dancing, sexual activity/competition (combined scale) and rowdiness were associated with increased intoxication. Of the five contextual variables analysed, only greater number of customers and later observation time were associated with higher intoxication. Non-significant variables (city, police outside the venue, outdoor drinking area) were excluded from further analyses. 

**Table 1:** Proportion of observations displaying environmental characteristics, and mean scale ratings for environmental measures, by city of observation.

		Liverpool	Palma	Utrecht	Ljubljana	P
**Number of venues**		15	15	15	15	
**Number of visits ^1^**		60	60	57	60	
**Venue entrance**						
Door staff	% Yes	98.3	88.3	75.4	63.3	<0.001
Queue	% Yes	15.0	35.0	31.6	13.3	0.006
Entrance fee	% Yes	11.7	40.0	14.0	26.7	0.001
House rules (entry)	% Yes	8.3	46.7	31.6	41.7	<0.001
**Physical environment**						
Seating	Mean	6.8	6.5	7.5	4.0	<0.001
Noise	Mean	6.2	6.5	5.8	5.1	<0.001
Crowding	Mean	4.7	3.9	5.1	3.7	0.001
Ventilation	Mean	2.1	3.6	3.6	2.4	<0.001
Temperature	Mean	4.2	4.7	5.4	4.4	<0.001
Clearing	Mean	4.8	4.8	6.6	4.4	<0.001
Glass on floor	Mean	2.5	1.6	2.5	1.4	0.006
Cleanliness	Mean	4.4	4.6	6.2	4.1	<0.001
Toilets	Mean	3.8	4.1	4.0	3.8	0.764
Lighting	Mean	3.1	4.2	3.6	2.8	<0.001
**Bar activities**						
Dance floor	% Yes	86.7	46.7	71.9	36.7	<0.001
Pool tables	% Yes	6.7	11.7	0.0	6.7	0.080
TV screens	% Yes	68.3	57.1	52.6	46.7	0.103
House rules (inside)	% Yes	3.3	38.3	12.3	63.3	<0.001
Rock/heavy music	% Yes	3.3	31.7	5.3	23.3	<0.001
Rap/hiphop music	% Yes	58.3	0.0	19.3	15.0	<0.001
Pop/dance music	% Yes	90.0	68.3	78.9	58.3	0.001
**Alcohol and food**						
Alcoholic drink promotions	% Yes	46.7	13.3	17.5	28.3	<0.001
Low drink prices ^2^	% Yes	37.9	73.3	66.7	36.7	<0.001
High alcohol drinks	% Yes	41.7	95.0	5.3	40.0	<0.001
Soft drink promotions	% Yes	1.7	21.7	21.1	15.0	0.007
Plastic glassware	% Yes	30.0	11.9	8.8	73.3	<0.001
Table service	% Yes	3.3	25.0	7.0	78.3	<0.001
Food service	% Yes	3.3	6.7	3.5	16.7	0.018
*Price of a bottle of lager (euros) ^3^*	Mean	3.81	4.18	2.28	2.89	<0.001
*Price of a glass of wine (euros)*	Mean	3.56	3.69	2.81	2.29	<0.001
*Price of a vodka and orange (euros)*	Mean	3.73	7.13	5.39	4.29	<0.001
*Price of a glass of coke (euros)*	Mean	1.69	3.65	2.10	2.02	<0.001

**^1 ^**Four visits were made to each venue with the exception of two venues in Utrecht, where only three visits were possible. One visit in Utrecht was excluded as no measurement of intoxication was recorded. ^2^ Based on the mean price of either lager or spirits depending on which drink was most commonly being consumed in the venue. ^3 ^Prices in Liverpool were converted from £ sterling to Euros at an exchange rate of 1.1531.

**Table 2:** Percentage of visits recording staffing and customer factors, and mean ratings for staffing and customer related scales, by city.

		Liverpool	Palma	Utrecht	Ljubljana	*P*
**Staff characteristics**						
Fewer bar staff	% Yes	16.7	70.0	38.6	10.0	<0.001
Young staff	% Yes	55.0	0.0	47.4	46.7	<0.001
Male staff	% Yes	48.3	26.7	73.7	60.0	<0.001
Glass collectors	% Yes	78.3	61.7	68.4	8.3	<0.001
**Staff behaviours**						
Staff monitoring	Mean	2.6	3.3	3.8	2.9	0.004
Staff coordination	Mean	4.2	5.0	4.7	3.8	0.002
Staff attitude	Mean	1.5	3.2	2.1	1.7	<0.001
Staff boundaries	Mean	1.3	3.4	3.4	1.6	<0.001
Permissiveness	Mean	2.9	1.8	2.4	0.9	<0.001
**Customer type**						
Male clientele	% Yes	60.0	75.0	63.2	81.7	0.033
Young clientele	% Yes	11.7	8.3	33.3	11.7	0.001
Single sex groups	% Yes	70.0	36.7	77.2	30.0	<0.001
**Customer behaviours**						
Dancing	Mean	4.5	3.7	4.8	3.3	0.033
Sexual activity	Mean	3.2	3.1	3.0	2.6	0.436
Sexual competition	Mean	3.5	2.7	2.7	1.7	<0.001
Rowdiness	Mean	3.3	2.9	3.2	0.9	<0.001
Movement	Mean	4.8	4.7	4.9	4.0	0.099
**Additional variables**						
Police outside	% Yes	33.3	18.3	7.3	1.7	<0.001
Outdoor area	% Yes	23.3	66.7	63.2	86.7	<0.001
100+ customers	% Yes	63.3	81.7	59.6	35.0	<0.001
Intoxication *	Mean	4.0	3.7	4.0	3.5	0.313

* Main variable of interest.

A multivariate analysis was conducted for each block of variables, with models also including customer number and observation time variables. Here, no venue entry characteristics were associated with intoxication ratings (Table 3). Within physical environment variables, greater movement/crowding (combined scale) and poorer washroom facilities were associated with higher ratings. The presence of a dance floor and TV screens were the only bar activity factors associated with intoxication. For alcohol and food service, promotion of non-alcoholic drinks and plastic glassware were associated with higher ratings and table service with lower ratings. Poorer staff monitoring and greater permissiveness were the only staff factors associated with higher intoxication. Customer factors associated with higher ratings were younger clientele, dancing, sexual activity/competition and rowdiness. 

**Table 3:** Hierarchical modelling: Associations between environmental characteristics and customer intoxication ratings.

				Multivariate					
		Bivariate		Block analysis		Model 1		Model 2	
	**Variable**	*Estimate*	*P*	*Estimate*	*P*	*Estimate*	*P*	*Estimate*	*P*
**Contextual variables #**	>100 customers	0.945	***			0.037	ns	0.139	ns
	Later visit	1.223	***			0.483	*	0.740	***
**Venue entrance**	Door staff	1. 017	**	0.496	ns				
	Queue	0.715	*	−0.229	ns				
	Entrance fee	0.823	*	0.124	ns				
	House rules (entry)	0.201	ns	0.142	ns				
**Physical environment**	Seating	0.240	***	0.062	ns				
	Noise level	0.282	***	0.060	ns				
	Movement/Crowding	0.191	***	0.087	*	0.025	ns	0.056	ns
	Ventilation/Lighting	0.280	***	0.092	ns				
	Temperature	0.380	***	0.058	ns				
	Clearing/Cleanliness	0.139	***	0.017	ns				
	Glass on floor	0.296	***	0.030	ns				
	Toilets	0.316	***	0.128	*	0.097	*	0.103	*
**Bar activities**	Dancefloor	1.252	***	0.993	***	0.269	ns	0.557	*
	Pool tables	−0.046	ns	−0.181	ns				
	TV screens	0.282	ns	0.569	*	0.107	ns	0.266	ns
	House rules (inside)	−0.132	ns	−0.093	ns				
	Rock/heavy music	−0.312	ns	−0.026	ns				
	Rap/hiphop music	0.080	ns	−0.217	ns				
	Pop/dance music	0.115	ns	−0.286	ns				
**Alcohol and food service**	Alcoholic drink promotions	0.297	ns	0.336	ns				
	Low drink prices	−0.350	ns	−0.344	ns				
	Soft drink promotions	0.888	**	0.833	**	0.631	*	0.690	**
	Plastic glassware	0.706	**	0.818	**	0.602	**	0.614	**
	Table service	−0.936	**	−0.882	**	0.031	ns	−0.090	ns
	Food service	−1.183	*	−0.394	ns				
**Venue staff**	Fewer bar staff	0.345	ns	−0.027	ns				
	Young staff	−0.084	ns	0.020	ns				
	Male staff	0.406	ns	0.202	ns				
	Glass collectors	0.539	*	0.235	ns				
	Staff monitoring	0.209	***	0.163	**	0.071	ns	0.081	ns
	Staff coordination	0.024	ns	−0.113	ns				
	Staff attitude	0.206	*	0.181	ns				
	Staff boundaries	0.130	*	0.052	ns				
	Permissiveness	0.526	***	0.425	***	0.160	*	0.298	***
**Customer factors**	Male clientele	−0.017	ns	−0.018	ns				
	Young clientele	0.886	**	0.590	*	0.316	ns		
	Single sex groups	0.089	ns	−0.081	ns				
	High alcohol drinks	0.181	ns	0.047	ns				
	Dancing	0.276	***	0.126	**	0.073	ns		
	Sexual activity/competition	0.237	***	0.085	*	0.065	*		
	Rowdiness	0.460	***	0.243	***	0.125	ns		

Analysis uses hierarchical modelling. # These two variables were included in all block analyses. ns = not significant; * *P* < 0.05; ** *P* < 0.01; *** *P* < 0.001. For significant associations in multivariate analyses, slope direction indicates whether the variable was associated with an increase or decrease (-) in intoxication rating.

All variables independently associated with intoxication ratings in block analyses were entered into an overall model (Model 1, Table 3), along with number of customers and observation timing. The model identified six factors independently associated with higher intoxication ratings: later observation time, poorer washroom facilities, non-alcoholic drink promotions, plastic glassware, greater permissiveness and higher customer sexual activity/competition. As customers will be attracted to venues based on their social and physical environments, a second model was constructed that excluded customer-focused variables. Here, all independent associations between non-customer factors and intoxication remained, and those with later observation timing, non-alcoholic drink promotions and permissiveness were strengthened. An independent relationship also emerged between intoxication ratings and the presence of a dance floor. 

## 4. Discussion

This study is among the first to explore associations between intoxication and environmental factors in European bars, and the first to do so cross-nationally. The study’s multi-country nature means findings may have been affected by structural and cultural factors, such as differences in licensing legislation and variation in the interpretation of bar characteristics and intoxication across research teams. To address this latter point, we used an established methodology [17,19] and a detailed training programme to develop consistency in measurement recording. Nevertheless, the relatively small variations seen between cities in ratings of intoxication may in part be due to variations in researchers’ cultural exposure and norms for what was considered drunk. Drink prices cannot be considered representative for each city, while drink serving sizes and strengths may have varied [21]. Further, as with all cross-sectional studies, we cannot ascertain causal relationships between bar characteristics and intoxication. However, our study does identify characteristics of bars where intoxication may be more likely, and consequently provides intelligence to inform bar-focused interventions to prevent alcohol-related harm. 

Several of our findings are consistent with research elsewhere. Many characteristics typically associated with alcohol-related harm (e.g., loud music, crowding, lack of seating) [20] were associated with intoxication in bivariate analysis, and some that were significant in multivariate analysis have been identified as risk factors elsewhere. For example, permissive bar environments, poor cleanliness (e.g., poorer washroom facilities) and measures of sexual competition have been associated with aggression and disorder in studies in Canada [17], Australia [22] and Scotland [12]. 

Other aspects of our findings are novel. Thus, this is the first observational study to identify associations between intoxication and both plastic glassware and promotion of non-alcohol drinks. Plastic glassware is widely used as a harm reduction measure in drinking premises, with the aim of preventing serious injuries following the use of glassware as a weapon [23,24]. In some countries its use can be mandated through licensing legislation. In Glasgow, Scotland, glass was banned in late night drinking venues in 2006. There were some exceptions, and a study found that disorder in bars that used only plastic glassware resulted in fewer injuries than that occurring in bars where glass was still used [24]. Plastic glassware can therefore help reduce injury in bars, yet does little to prevent violence nor, as our study indicates, the intoxication that drives this. Thus, use of plastic glassware should not be considered sufficient to demonstrate responsible management; its use must be accompanied by action to reduce intoxication in order to prevent broader alcohol-related harms, including those that can occur when intoxicated individuals leave the relative safety of glass-free premises [25]. 

A more surprising finding was the association between non-alcoholic drink promotions and higher intoxication ratings. There are several possibilities for this. Firstly, as with plastic glassware, the promotion of non-alcoholic drinks may reflect a concerted effort in problematic premises to reduce harm. Another explanation may relate to modern drinking patterns. A survey conducted alongside this study found high levels of preloading among nightlife users in the four cities [7]. With many customers entering bars after having already consumed significant quantities of alcohol, venue managers may consider non-alcoholic drinks to provide greater potential for sales; particularly legal sales since service of alcohol to intoxicated individuals is often illegal. Preloading may also account for the lack of association between intoxication and cheap alcoholic drink promotions, lower alcohol prices or high alcohol content drinks. However, the most plausible explanation might be provided by the fact that many non-alcoholic drinks promoted were “energy” drinks (e.g., containing caffeine). These drinks are commonly used as mixers with spirits, can desensitise users to the symptoms of intoxication, can have diuretic effects that can increase thirst, and are used as stimulants by nightlife users to help them stay awake and continue drinking over long nights [26,27]. Bars may exploit these effects and promote energy drinks to encourage customers to continue purchasing and consuming drinks. Numerous studies have identified increased risks of intoxication and alcohol-related problems among individuals that consume alcohol mixed with energy drinks [28,29,30]. Any efforts to promote non-alcoholic drinks in bars as a preventive measure should be implemented with caution, and should specifically exclude energy drinks. 

In line with customer behaviour reflecting bar policy, after customer-focused variables were removed from analyses the relationship between permissive environments and intoxication was strengthened. Bars that tolerate intoxication and raucous behaviour are likely to attract individuals who want to get drunk and behave in ways that may prevented elsewhere. Among other management-focused variables only poor washroom facilities, a potential marker of staff negligence, was associated with intoxication in our final models. However, all physical environment characteristics showed strong associations with intoxication before interactions between them were controlled for. This indicates that factors such as inadequate glass clearing, poor cleanliness, and poor ventilation and lighting cluster in high risk bars, suggesting a general lack of managerial care in such premises. Thus, while poor physical environments may not cause intoxication per se, they could be considered as a syndrome diagnostic of venues where intoxication and harm is likely. The development of standards for licensed premises is recommended through international alcohol strategies [1,2]. However, evidence for the effectiveness of such measures as standalone interventions is scant [31]. Where management-focused interventions have shown success they have typically been backed up by strong enforcement and packaged within multi-agency programmes [15,31,32,33]. The importance of enforcing and monitoring licensing legislation is also recognised in international strategies. Ensuring such activity is implemented alongside measures to train staff and develop standards should be considered imperative.

Professionally-managed bars have the potential to reduce drunkenness and so contribute to both safer drinking environments and public health. Venue staff can control access to alcohol, manage confrontation, provide environments where abusive behaviour is not tolerated, and offer customer care services. Whilst we have identified the potential impacts of poor bar management, other drinking environments (e.g., private parties, public spaces) offer little opportunity for managing drinkers’ behaviour and safety. Recent years have seen a trend in Europe towards reduced alcohol sales in on-trade premises and increased sales in supermarkets and shops for consumption in private settings, driven largely by cheaper off-sales prices [34]. In the longer term, providing well-managed environments where people can socialise safely may be a more sustainable strategy for professional bar operators than focusing purely on selling large quantities of alcohol. Whilst strategies should aim to create well-managed bars that do not permit drunkenness, such practices are likely to be helped by regulation that prevents the sale of cheap alcohol elsewhere. 

## 5. Conclusions

Preventing harm in drinking environments requires interventions that recognise and address the contributors to intoxication. Consistent with international research, our study suggests that venues where intoxication occurs can have a clustering of “bad” environmental features that manifest through poor managerial care. The variables with the strongest relationships with intoxication ratings were permissiveness (identified as a general indifference towards patrons’ behaviours) and later observation time. Thus, permissive late night venues are likely to attract individuals who want to get (or are already) drunk and provide environments with few behavioural expectations. In such venues, harm reduction measures such as plastic glassware can be common, implemented specifically to prevent intoxicated aggression turning into serious injury. These measures may be tokenistic; having little impacts on sales and profits and being relatively easy for venues to adopt, whether to demonstrate social responsibility or meet licensing requirements. However, they do little to address the root causes of harm. Our findings suggest that greater focus on managerial practice is needed. All features of the physical, social and staffing environment within bars stem from management decisions, including how venues are designed, how staff are trained, and how customers are permitted to behave. In some circumstances, attracting heavy drinking patrons may represent a commercially attractive model despite the poor health and anti-social outcomes associated with drunkenness. While many establishments may be well placed to adopt recognised managerial standards some of the most risky will only change when faced with regulation and enforcement. 

## Appendix

**Table A1:** Description of observational schedule measurements used in analyses.

Scale variables			Categorical variables	
Label	Scale	Scale range	Label	Yes/No
Intoxication *	Intoxication level of people in the venue	0 no sign of intoxication 9 → everyone is drunk	Door staff	Staff managing entrance to the venue
Seating	Proportion of the venue floor space containing seating	0 90% or more → 9 <10%	Queue	There was a queue to enter the venue
			Entrance fee	Entrance fee had to be paid
Noise	Noise level in loudest part of venue	0 very quiet/easy to talk → 9 hurts ears/cannot talk	House rules (entry)	House rules displayed at venue entrance
Crowding ^a^	Crowding at busiest time (exc.dancefloor)	0 lots of space → 9 cannot move	Dance floor	Venue had a designated dance floor area
Movement ^a^	Movement (at busiest time/part of venue)	0 little movement → 9 constant	Pool tables	Venue had pool tables
Ventilation ^b^	Ventilation in the venue	0 extremely fresh → 9 extremely stuffy/stale	TV screens	Television screens ^g^ visible in the venue
Lighting ^b^	Level of lighting inside the venue	0 bright/can clearly see → 9 very dark/can hardly see	House rules (venue)	House rules displayed inside the venue
Temperature	Temperature in the venue	0 very cold → 9 very warm	Rock/heavy music	Rock/heavy metal music being played
Clearing ^c^	Clearing of tables/other surfaces ^e^	0 always → 9 never	Rap/hip hop music	Rap or hip hop music being played
Cleanliness ^c^	Extent that indoor premises are kept clean (spills, litter) including the floor	0 always → 9 never	Pop/dance music	Pop or dance music being played
			Alcoholic drink promotions	Cheap drink promotions ^h^ offered
Glass on floor	Extent of glass/bottles on venue floor^f^	0 none → 9 everywhere	Low drinks prices	Drink prices below average for that city ^i^
Toilets	Extent that toilets are kept in order (e.g., locks) and stocked (soap, toilet rolls *etc.*)	0 clean/fresh/stocked → 9 vandalised/foul	Soft drink promotions	Non-alcoholic drinks promoted ^j^
			Plastic glassware	Drinks served in plastic glasses ^k^
Staff monitoring	To what extent are staff generally monitoring all areas of the venue?	0 constantly monitored → 9 unmonitored	Table service	Drinks served at tables
			Food service	Food available during the observation
Staff coordination	To what extent do staff seem to be coordinated as a team?	0 constant radio or eye contact → 9 not coordinated at all	Fewer bar staff	30 or more customers per bar server
			Young staff	>50% thought to be under age 25
Staff attitude	Are servers cheerful, courteous and friendly (CCF) in a professional way or distant, unfriendly, stern or even rude/obnoxious (DUS)?	0 all were CCF → 9 all were DUS	Male staff	>50% male
			Glass collectors	Glass collectors working in the venue
			Male clientele	>50% clientele were male
Staff boundaries	Extent that servers maintained professional (P) boundaries from patrons	0 all completely P, clear boundaries → all socialising with customers	Young clientele	>50% clientele estimated to be <age 22
			Single sex groups	>50% clientele in single sex groups
**Scale variables**			**Categorical variables**	
**Label**	**Scale **	**Scale range**	**Scale **	**Label**
Permissiveness	Overall decorum /behavioural expectations	0 no offensive/abusive behaviour → 9 anything goes	High alcohol drinks	High alcohol content ^l ^drinks most common
			Police outside	Police were outside the venue at entry
Dancing	Proportion of customers dancing	0 <10% → 9 90% or more	Outdoor area	Outdoor eating/drinking/smoking area
Sexual activity ^d^	Sexual activity in venue	0 none → 9 explicit sexual contact	100+ customers	100+ customers in venue at peak time
Sexual competition ^d^	Sexual competition in venue	0 scoping not the focus for anyone → 9 scoping the focus of 76-100%	Later visit	Later 50% of observations (per city)
			Rowdiness	Global rating of rowdiness in the venue	0 none/very rare → 9 out of control		

* Main variable of interest. The following variables were strongly correlated and were combined into single scales measured from 0 to 18: ^a^ Crowding and movement (r = 0.686; cronbach’s alpha 0.813); ^b^ Ventilation and Lighting (r = 0.607; cronbach’s alpha 0.755); ^c^ Clearing and Cleanliness (r = 0.788; cronbach’s alpha 0.881); ^d^ Sexual activity and Sexual competition (r = 0.765; cronbach’s alpha 0.866); ^e^ Highest rating from two scales covering tables/other surfaces separately; ^f ^Highest rating from two scales covering glass/bottles separately; ^g^ Typically showing music videos or venue marketing/promotions; ^h^ e.g., buy one get one free, free shots; ^i^ Based on spirits or lager depending on which drink was most commonly being consumed in the venue; ^j^ Including energy drinks; ^k^ Partly or wholly; ^l^ High alcohol: spirits/wine, low alcohol: lager/cider/alcopops.

## References

[1] World Health Organization (2010). Global Strategy to Reduce the Harmful Use of Alcohol. http://www.who.int/substance_abuse/activities/gsrhua/en/.

[2] World Health Organization Regional Office for Europe (2011). European Action Plan to Reduce the Harmful Use of Alcohol 2012–2020.

[3] Gmel G., Heeb J.L., Rezny L., Rehm J., Kuo-Mohler M. (2005). Drinking patterns and traffic casualties in Switzerland: Matching survey data and police records to design preventive action. Public Health.

[4] Rowe S.C., Wiggers J.H., Wolfenden L., Francis J.L. (2010). Establishments licensed to serve alcohol and their contribution to police-recorded crime in Australia: Further opportunities for harm reduction. J. Stud. Alcohol Drugs.

[5] Bellis M.A., Hughes K., Quigg Z., Morleo M., Jarman I., Lisboa P. (2010). Cross-sectional measures and modelled estimates of blood alcohol levels in UK nightlife and their relationships with drinking behaviours and observed signs of inebriation. Subst. Abuse Treat. Prev. Policy.

[6] Clapp J.D., Reed M.B., Win J.W., Shillington A.M., Croff J., Holmes M.R., Trim R.S. (2009). Blood alcohol concentrations among bar patrons: A multi-level study of drinking behaviour. Drug Alcohol Depend..

[7] Hughes K., Quigg Z., Bellis M.A., van Hasselt N., Calafat A., Kosir M., Juan M., Duch M., Voorham L. (2011). Drinking behaviours and blood alcohol concentration in four European drinking environments: A cross-sectional study. BMC Public Health.

[8] Livingston M., Chikritzhs T., Room R. (2007). Changing the density of alcohol outlets to reduce alcohol-related problems. Drug Alcohol Rev..

[9] Grubesic T.H., Pridemore W.A. (2011). Alcohol outlets and clusters of violence. Int. J. Health Geogr..

[10] Livingston M. (2011). Alcohol outlet density and harm: Comparing the impacts on violence and chronic harms. Drug Alcohol Rev..

[11] Homel R., Carvolth R., Hauritz M., McIlwain G., Teague R. (2004). Making licensed premises safer for patrons: What environmental factors should be the focus of interventions?. Drug Alcohol Rev..

[12] Forsyth A.J.M. Assessing the Relationships between Late Night Drinks Marketing and Alcohol-Related Disorder in Public Space. http://alcoholresearchuk.org/2007/04/06/relationships-between-late-night-drinks-marketing-and-alcohol-related-disorder/.

[13] Graham K., Osgood D.W., Wells S., Stockwell T. (2006). To what extent is intoxication associated with aggression in bars? A multilevel analysis. J. Stud. Alcohol.

[14] Newton A.D., Hirschfield A. (2009). Measuring violence in and around licensed premises: The need for a better evidence base. Crime Prevent. Commun. Saf..

[15] Graham K., Homel R.  (2008). Raising the Bar: Preventing Aggression in and Around Bars, Pubs and Clubs.

[16] Madensen T.D., Eck J.E. (2008). Violence in bars: Exploring the impact of place manager decision-making. Crime Prevent. Commun. Saf..

[17] Graham K., Bernard S., Osgood D.W., Wells S. (2006). Bad nights or bad bars? Multi-level analysis of environmental predictors of aggression in late-night large-capacity bars and clubs. Addiction.

[18] Green J., Plant M.A. (2007). Bad bars: A review of risk factors. J. Subst. Abuse.

[19] Graham K., La Rocque L., Yetman R., Ross T.J., Guistra E. (1980). Aggression and barroom environments. J. Stud. Alcohol.

[20] Hughes K., Quigg Z., Eckley L., Bellis M.A., Jones L., Calafat A., Kosir M., van Hasselt N. (2011). Environmental factors in drinking venues and alcohol-related harm: The evidence base for European intervention. Addiction.

[21] Gual A., Martos A.R., Lligona A., Llopis J.J. (1999). Does the concept of a standard drink apply to viticultural societies?. Alcohol Alcohol..

[22] Homel R., Clark J. (1994). The Prediction and Prevention of Violence in Pubs and Clubs. Crime Prevention Studies.

[23] Anderson Z., Whelan G., Hughes K., Bellis M.A. Evaluation of the Lancashire Polycarbonate Glass Pilot Project. http://www.cph.org.uk/showPublication.aspx?pubid=561.

[24] Forsyth A.J.M. (2008). Banning glassware from nightclubs in Glasgow (Scotland): Observed impacts, compliance and patron’s views. Alcohol Alcohol..

[25] Bellis M.A., Hughes K. (2011). Getting drunk safely? Night-life policy in the UK and its public health consequences. Drug Alcohol Rev..

[26] Ferreira S.E., De Mello M.T., Pompeia S., De Souza-Formigoni M.L.O. (2006). Effects of energy drink ingestion on alcohol intoxication. Alcohol Clin. Exp. Res..

[27] Marczinski C.A. (2011). Alcohol mixed with energy drinks: Consumption patterns and motivations for use in U.S. college students. Int. J. Environ. Res. Public Health.

[28] Thombs D.L., O’Mara R.J., Tsukamoto M., Rossheim M.E., Weiler R.M., Merves M.L., Goldberger B.A. (2010). Event-level analyses of energy drink consumption and alcohol intoxication in bar patrons. Addict. Behav..

[29] O’Brien M.C., McCoy T.P., Rhodes S.D., Wagoner A., Wolfson M. (2008). Caffeinated cocktails: Energy drink consumption, high-risk drinking, and alcohol-related consequences among college students. Acad. Emerg. Med..

[30] Brache K., Stockwell T. (2011). Drinking patterns and risk behaviors associated with combined alcohol and energy drink consumption in college drinkers. Addict. Behav..

[31] Jones L., Hughes K., Atkinson A.M., Bellis M.A. (2011). Reducing harm in drinking environments: A systematic review of effective approaches. Health Place.

[32] Wallin E., Norstrom T., Andreasson S. (2003). Alcohol prevention targeting licensed premises: A study of effects on violence. J. Stud. Alcohol.

[33] Warpenius K., Holmila M., Mustonen H. (2010). Effects of a community intervention to reduce the serving of alcohol to intoxicated patrons. Addiction.

[34] Hughes K., Bellis M.A., Anderson P., Moller L., Galea G. (2012). Drinking Environments. Alcohol in the European Union: Consumption, Harm and Policy Approaches.

